# Ester-Bond-Cleavable Self-Degradable Gel Particles for Temporary Plugging and Controlled Deplugging in Multi-Fracture Reservoir Systems

**DOI:** 10.3390/molecules31111979

**Published:** 2026-06-05

**Authors:** Zhe Li, Yaguang Qu, Li Han, Gang Wang

**Affiliations:** 1 College of Petroleum Engineering, Yangtze University, Wuhan 430100, China; 2 PetroChina Huabei Oilfield Company, Renqiu 062552, China; 3 State Key Laboratory of Low Carbon Catalysis and Carbon Dioxide Utilization, Yangtze University, Wuhan 430100, China; 4 The Sixth Oil Production Plant, Changqing Oilfield Branch, Xi’an 710018, China

**Keywords:** ester-bond-cleavable gel particles, self-degradable gel, temporary plugging, flow diversion, multi-fracture reservoir

## Abstract

Temporary plugging and flow diversion in multi-fracture reservoirs require gel particles that can provide stable plugging in dominant fractures while enabling low-residue deplugging after treatment. In this study, ester-bond-cleavable self-degradable gel particles were prepared by aqueous free-radical crosslinking using acrylamide, 2-acrylamido-2-methylpropanesulfonic acid, N-vinyl-2-pyrrolidone, and polyethylene glycol diacrylate as a hydrolysable crosslinker. An MBAA-crosslinked particle was used as a nondegradable control. FTIR and SEM results confirmed the formation of ester-containing crosslinked networks with tunable morphology. Among the prepared samples, EGP-2 showed a balanced hydration and mechanical response, with an equilibrium swelling ratio of 7.12 g/g at 80 °C and 100,000 mg/L salinity, a storage modulus of 205 Pa at 1 Hz, a compressive stress of 54.2 kPa at 30% strain, and a height recovery ratio of 91.8%. In single-fracture tests, EGP-2 achieved plugging efficiencies of 98.6% and 97.4% in 0.5 and 1.0 mm fractures, respectively, with corresponding erosion retention ratios of 94.1% and 92.6%. In a three-parallel-fracture model, EGP-2 reduced the dominant-fracture flow split ratio from 78.4% to 32.8%, while increasing the combined flow split ratio of the medium and narrow fractures from 21.6% to 67.2%, corresponding to a flow diversion efficiency of 58.2%. After aging at 120 °C for 96 h, EGP-2 exhibited a mass loss ratio of 78.4% and a G′ retention ratio of 25.4%. Subsequent flowback tests showed a flowback ratio of 82.6%, a permeability recovery ratio of 88.7%, and a residue ratio of 10.9%. These results demonstrate that ester-bond-cleavable gel particles can integrate temporary plugging, flow diversion, and controlled deplugging, offering a low-residue strategy for multi-fracture reservoir conformance control.

## 1. Introduction

Fractured reservoirs commonly contain complex fracture networks with strong heterogeneity in fracture aperture, connectivity, and flow resistance. During water flooding, gas injection, chemical flooding, or fracturing-fluid displacement, injected fluids preferentially enter highly conductive fractures or dominant channels, while medium and narrow fractures remain insufficiently swept [1,2,3]. This preferential flow behavior causes early water breakthrough, poor sweep efficiency, and ineffective utilization of remaining oil or gas in less-connected fracture regions. Therefore, temporary plugging and flow diversion have become important strategies for improving fluid redistribution in fractured reservoirs. An effective temporary plugging material should not only establish sufficient flow resistance in dominant fractures, but also maintain injectability, deformability, and adaptability to fracture-scale variations [4,5]. More importantly, after the target flow-diversion stage, the plugging material should be removable or degradable to avoid long-term residue retention and conductivity damage.

Gel-based plugging materials have attracted extensive attention because of their water-swelling capacity, viscoelastic deformability, and ability to form bridge-packing structures in fracture channels [6,7,8]. Compared with rigid particles, gel particles can deform under fracture-wall confinement and fluid displacement, thereby improving their ability to migrate into fractures, bridge at local constrictions, and form compacted plugging zones [9,10]. Crosslinked polymer gel particles based on acrylamide, sulfonated monomers, and other hydrophilic units have been widely investigated for conformance control, profile modification, and temporary plugging applications [11,12,13]. Their swelling behavior and mechanical stability can be adjusted by monomer composition, crosslinking density, particle size distribution, and salinity- or temperature-responsive structural units [14,15]. However, conventional gel particles are usually designed to maximize plugging strength and erosion resistance, while their post-treatment removal behavior is often insufficiently considered. Strongly crosslinked particles may remain in fractures after treatment, causing residual blockage and reducing fracture conductivity.

Self-degradable gel systems provide a potential route to resolve the conflict between plugging stability and deplugging recovery. By introducing cleavable chemical bonds into the crosslinked network, gel particles can retain mechanical integrity during injection and temporary plugging, while gradually undergoing network attenuation under reservoir conditions [16,17,18]. Among different cleavable linkages, ester bonds are particularly attractive because they can undergo hydrolysis in aqueous environments, and their cleavage rate can be regulated by temperature, pH, salinity, and molecular environment [19,20]. Polyethylene glycol diacrylate (PEGDA) contains ester-containing crosslinking structures and can participate in free-radical polymerization to form hydrolysable networks. Incorporating PEGDA into acrylamide-based gel particles may therefore provide both elastic crosslinking and controllable degradation. Nevertheless, the introduction of cleavable crosslinking structures must be carefully balanced. A low crosslinking density may accelerate swelling and degradation but reduce plugging stability, whereas excessive crosslinking may improve strength but restrict swelling, deformability, and post-treatment removal.

Another challenge lies in the evaluation system itself. Many temporary plugging studies are still based on single-fracture models, where plugging pressure, plugging efficiency, and erosion resistance are used as primary indicators [21,22]. Although such tests are necessary for evaluating basic plugging performance, they do not fully represent flow competition in multi-fracture reservoirs. In a real fracture network, injected particles must preferentially enter dominant fractures, increase their flow resistance, and force subsequent fluid into medium and narrow fractures. Therefore, the key performance should not only include plugging efficiency in a single fracture, but also selective plugging, flow split redistribution, flow diversion efficiency, deplugging behavior, flowback recovery, and residue control in a multi-fracture system. A material that generates high plugging pressure but cannot degrade or flow back may not be suitable for reservoir applications requiring temporary and recoverable diversion.

Although degradable gel particles and PEGDA-based degradable networks have been reported previously, most existing studies have mainly focused on degradation-time regulation, gel strength, plugging pressure, or core-scale damage reduction. In such systems, PEGDA or other cleavable crosslinkers are generally used to introduce hydrolysable network junctions, whereas the coupling among degradable crosslinking chemistry, particle-scale deformability, selective plugging in competing fractures, flow split redistribution, and low-residue flowback has not been fully clarified. Therefore, the novelty of the present work does not lie simply in using PEGDA as a hydrolysable crosslinker, but in constructing an ester-bond-cleavable particle system and evaluating its complete plugging–diversion–deplugging sequence in a heterogeneous multi-fracture model. By comparing the PEGDA-crosslinked EGP series with an MBAA-crosslinked nondegradable control, this study clarifies how crosslinking chemistry regulates swelling, mechanical stability, fracture adaptability, degradation-assisted deplugging, and residue control. Based on these considerations, ester-bond-cleavable self-degradable gel particles were designed in this work for temporary plugging and controlled deplugging in multi-fracture systems. Acrylamide (AAm), 2-acrylamido-2-methylpropanesulfonic acid (AMPS), and N-vinyl-2-pyrrolidone (NVP) were used to construct a hydrophilic polymer framework, while PEGDA was introduced as a hydrolysable crosslinker to provide ester-bond-cleavable network sites. An MBAA-crosslinked particle system was prepared as a nondegradable control. The chemical structure, morphology, particle size distribution, hydration swelling, rheological behavior, compression stability, degradation response, single-fracture plugging performance, multi-fracture flow diversion, and deplugging recovery were systematically investigated. The objective was to clarify how ester-bond crosslinking density regulates the balance among plugging strength, flow diversion, and low-residue recovery, and to establish a plugging–diversion–deplugging mechanism for self-degradable gel particles in heterogeneous multi-fracture systems. This integrated evaluation framework provides a clearer distinction from previously reported degradable gel particles or PEGDA-based gels that are mainly assessed through bulk degradation, plugging strength, or single-channel flow resistance.

## 2. Results and Discussion

### 2.1. Structural Characterization of Ester-Bond-Cleavable Gel Particles

#### 2.1.1. FTIR Characterization

**Figure 1:**
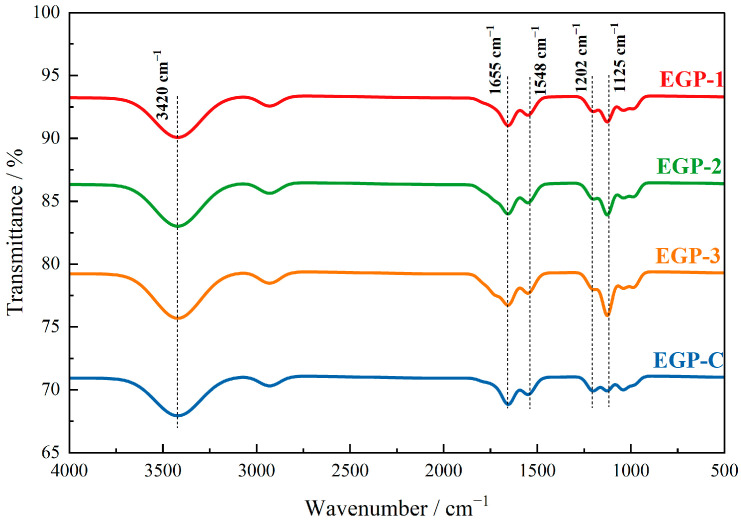
FTIR spectra of EGP-1, EGP-2, EGP-3, and EGP-C gel particles.

FTIR spectra of dry EGP-1, EGP-2, EGP-3, and EGP-C particles were recorded according to the procedure described in Section 4.4.9 to verify the incorporation of PEGDA-related crosslinking structures and to compare the chemical differences between ester-bond-cleavable particles and the MBAA-crosslinked control. Because the main band assignments are already summarized in Table 1, the following discussion focuses on the structural information most relevant to the subsequent degradation and deplugging behavior, rather than repeating each spectral assignment in detail. The FTIR spectra of different samples are shown in Figure 1, and the main band assignments are summarized in Table 1.

All samples showed the characteristic N–H/O–H, amide I, amide II, and S=O absorption bands near 3420, 1655, 1548, and 1202 cm^−1^, respectively. These common bands confirm the formation of an AAm/AMPS/NVP-based hydrophilic polymer framework in both the EGP series and EGP-C. Since the main monomer composition was identical among the samples, the similar backbone-related bands indicate that their later performance differences were mainly governed by the crosslinking structure rather than by changes in the principal polymer backbone. Compared with EGP-C, EGP-1, EGP-2, and EGP-3 exhibited more evident PEGDA-related absorption features, including the C–O–C stretching band near 1125 cm^−1^ and a weak shoulder or enhanced absorption in the 1700–1740 cm^−1^ region. The latter can be associated with ester carbonyl C=O stretching, although partial overlap with the amide I band should be considered. Therefore, PEGDA incorporation was not judged from the ester-carbonyl region alone, but from the combined enhancement of the C–O–C and ester-carbonyl-related signals. This combined evidence supports the participation of PEGDA-derived ester-containing crosslinks in the EGP network.

EGP-C showed amide- and sulfonic-group-related bands similar to those of the EGP samples, but it lacked the PEGDA-dosage-dependent enhancement observed in the EGP series. This contrast indicates that EGP-C and the EGP samples shared a similar hydrophilic backbone but differed in the chemical nature of their crosslinking points. The EGP series contained PEGDA-derived ester-bearing junctions that could serve as hydrolysable sites under high-temperature aqueous conditions, whereas EGP-C was mainly stabilized by MBAA-derived nonhydrolysable covalent crosslinks. Thus, the FTIR results establish the structural basis for interpreting the later differences in swelling, mechanical stability, self-degradation, and controlled deplugging performance.

#### 2.1.2. SEM Morphological Characterization

**Figure 2:**
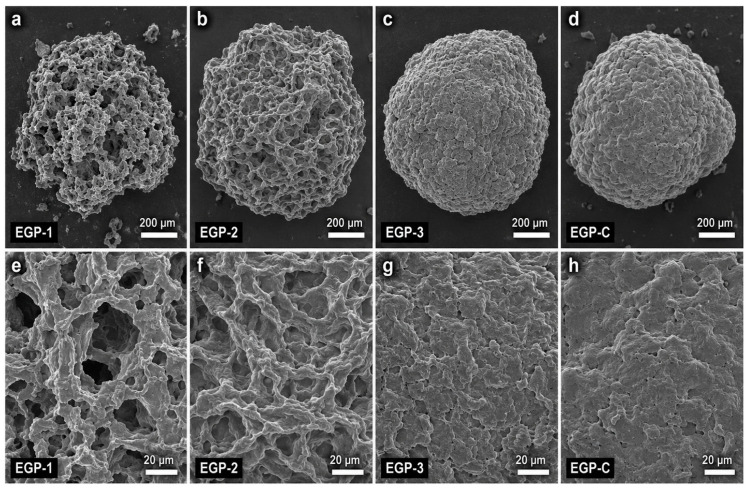
Low- and high-magnification SEM images of EGP-1, EGP-2, EGP-3, and EGP-C gel particles. (**a**–**d**) Low-magnification images of EGP-1, EGP-2, EGP-3, and EGP-C; (**e**–**h**) corresponding high-magnification images.

According to the method described in Section 4.4.10, SEM observations were conducted on dry gel particles and freeze-dried hydrated samples of EGP-1, EGP-2, EGP-3, and EGP-C to analyze the influence of different crosslinking structures on particle surface morphology and network compactness. The SEM analysis was used not only to describe particle morphology, but also to clarify how PEGDA dosage regulated the balance between surface openness and structural integrity. The SEM images of different samples are shown in Figure 2.

As shown in Figure 2a–d, all four gel particle samples exhibited irregular particle-like or quasi-spherical morphologies, indicating that the bulk gels could be converted into particle systems suitable for subsequent injection and fracture plugging experiments post-treatment. However, clear differences in surface compactness and pore openness were observed among the samples, reflecting the influence of crosslinking density on network formation during gel preparation and drying.

EGP-1 showed a loose surface with fragmented edges and open cavities, suggesting insufficient skeleton support at low PEGDA dosage. This structure may favor rapid water penetration, but it also makes the particles more susceptible to deformation or fragmentation under displacement. In contrast, EGP-3 exhibited a more compact surface with fewer open pores and clearer particle contours, indicating stronger network constraints at higher PEGDA dosage. Such compactness can improve particle integrity, but it may also restrict water uptake and reduce deformability during fracture migration.

EGP-2 presented a more balanced morphology. Its surface retained moderate pore openness while maintaining a relatively continuous wrinkled skeleton. This morphology is important for temporary plugging because pore openness facilitates hydration and elastic deformation, whereas skeleton continuity supports particle bridging, packing, and erosion resistance within fractures. Therefore, the SEM results provide a morphological explanation for why EGP-2 was expected to show a better balance between swelling adaptability and mechanical retention than EGP-1 or EGP-3.

EGP-C, as the MBAA-crosslinked nondegradable control sample, showed certain morphological differences from the PEGDA-gradient samples. It exhibited a relatively compact surface and less pronounced wrinkled structure than EGP-1 and EGP-2. Combined with the FTIR results, this contrast indicates that EGP-C and the EGP series had comparable hydrophilic backbones but different crosslinking structures, which further affected the resulting network morphology. Overall, SEM observations confirm that PEGDA dosage regulated the transition from a loose, open network to a compact, constrained network. This structural transition provides a morphological basis for interpreting the hydration, deformation, and fracture-retention behavior discussed in the following sections.

#### 2.1.3. Particle Size Distribution

**Figure 3:**
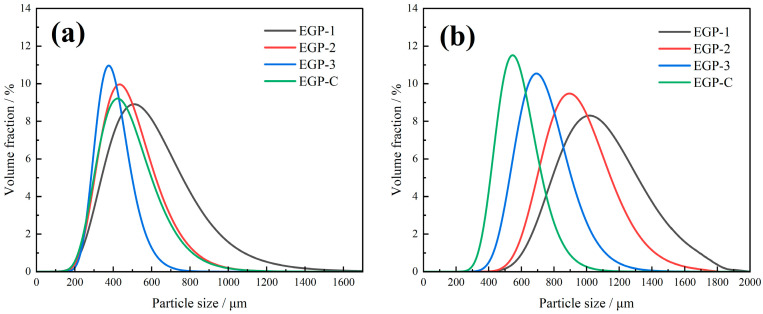
Particle size distribution curves of different gel particles. (**a**) Particle size distribution curves of dry gel particles; (**b**) particle size distribution curves of hydrated gel particles.

According to the particle size distribution method described in Section 4.4, the particle size distributions of dry and hydrated EGP-1, EGP-2, EGP-3, and EGP-C particles were measured to evaluate the size concentration of gel particles after grinding and sieving, as well as their hydration-induced size response in simulated formation water. Figure 3a,b show the particle size distribution curves of dry and hydrated gel particles, respectively.

As shown in Figure 3a, all four dry gel particle samples exhibited unimodal distributions, with the main particle size range concentrated within approximately 250–800 μm. This result indicates that grinding and sieving produced injectable particle populations with relatively controlled size ranges. However, the curve width varied among samples, suggesting that the crosslinking structure affected particle fragmentation during post-treatment. EGP-1 showed a broader dry-size distribution, consistent with its weaker skeleton support at low PEGDA dosage. In contrast, EGP-3 exhibited a narrower distribution, indicating that higher PEGDA crosslinking density improved dry-particle integrity. This trend agrees with the SEM observation that stronger network compactness reduced edge fragmentation during mechanical processing.

After hydration, the particle size distribution curves of all samples shifted toward larger particle sizes, indicating obvious water-absorption-induced swelling in simulated formation water. The extent of this shift was governed by the balance between water uptake and network constraint. EGP-1 showed the most pronounced size increase and the broadest hydrated distribution, reflecting strong swelling but reduced size uniformity. EGP-3 and EGP-C showed more limited hydrated size growth, which can be attributed to stronger network constraints that restricted water penetration and chain expansion. These two cases represent opposite particle responses: excessive swelling may weaken size control, whereas excessive constraint may limit deformation and fracture adaptability.

EGP-2 showed a more suitable hydrated particle-size response, with the main hydrated peak located between those of EGP-1 and EGP-3 and without the excessive broadening observed for EGP-1. This behavior indicates that moderate PEGDA crosslinking density allowed sufficient hydration expansion while maintaining particle-size concentration. For fracture plugging, this balance is more relevant than the absolute particle size alone. Particles must be large and deformable enough to contact fracture walls and form bridges, but they also need a stable size distribution to avoid premature inlet plugging or uncontrolled passage through wider channels. Therefore, the particle-size characteristics of EGP-2 provide a particle-scale basis for its subsequent single-fracture plugging and multi-fracture flow-diversion behavior.

### 2.2. Hydration Swelling Behavior Under Reservoir Conditions

**Figure 4:**
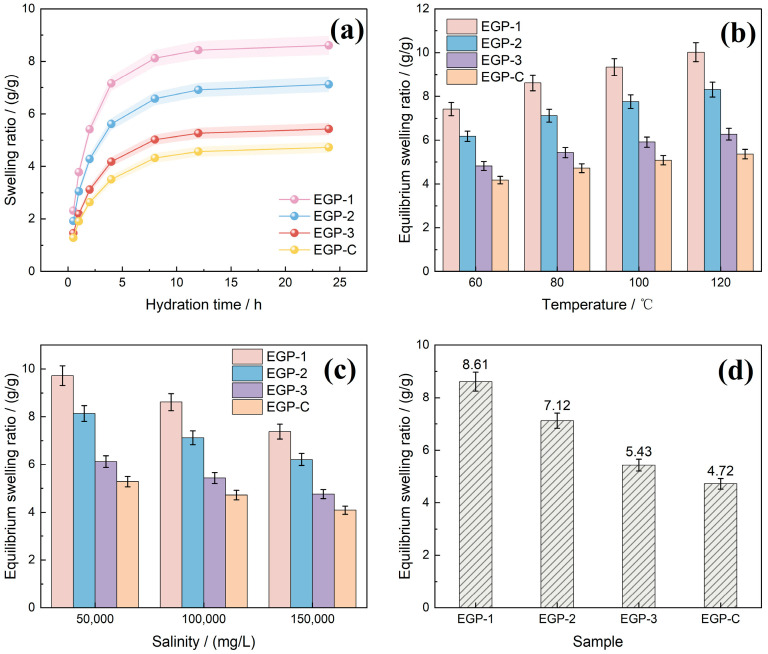
Hydration swelling behavior of gel particles under different conditions. (**a**) Swelling ratio as a function of hydration time at 80 °C and 100,000 mg/L salinity; (**b**) effect of temperature on equilibrium swelling ratio; (**c**) effect of salinity on equilibrium swelling ratio; (**d**) comparison of equilibrium swelling ratios of different samples. Error bars represent standard deviations, *n* = 3.

According to the hydration swelling test described in Section 4.4, the swelling behavior of EGP-1, EGP-2, EGP-3, and EGP-C was evaluated under different hydration times, temperatures, and salinities to clarify the influence of ester-bond crosslinking density on the hydration response and reservoir-environment adaptability of gel particles. Figure 4 summarizes the time-dependent swelling behavior, temperature response, salinity response, and equilibrium swelling capacity of the four samples. These results connect the hydrated particle-size shift discussed in Section 2.1.3 with the deformation and bridging behavior required for fracture plugging.

As shown in Figure 4a, all samples underwent rapid initial hydration followed by a gradual approach to equilibrium, reflecting water diffusion, network expansion, and elastic constraint within the gel particles. At 24 h, the equilibrium swelling ratios of EGP-1, EGP-2, EGP-3, and EGP-C were approximately 8.6, 7.1, 5.4, and 4.7 g/g, respectively, following the order EGP-1 > EGP-2 > EGP-3 > EGP-C. This order shows that lower PEGDA crosslinking density promoted water penetration and chain extension, whereas higher crosslinking density increased network constraints and suppressed volumetric expansion. The differences in equilibrium swelling ratio among the four samples were statistically significant (*p* < 0.05), confirming that crosslinking structure had a measurable effect on water uptake.

The differences among the EGP samples are more informative than the swelling values alone. EGP-1 showed the highest swelling ratio because its lower crosslinking density provided greater free volume and chain mobility; however, this strong swelling was accompanied by the broader hydrated particle-size distribution discussed in Section 2.1.3, implying weaker size control after hydration. EGP-3 exhibited restricted swelling due to stronger PEGDA-imposed constraints, which may limit deformation and fracture adaptability. EGP-2 maintained an intermediate swelling ratio, indicating that moderate PEGDA crosslinking allowed sufficient water uptake without excessive structural relaxation. EGP-C showed the lowest swelling capacity, consistent with the more constrained MBAA-crosslinked network.

Temperature had a clear influence on the hydration swelling behavior of gel particles. As shown in Figure 4b, the equilibrium swelling ratios of all samples generally increased as the temperature increased from 60 to 120 °C. For EGP-2, the equilibrium swelling ratio increased from approximately 6.2 g/g at 60 °C to approximately 8.3 g/g at 120 °C. This temperature-enhanced swelling can be attributed to increased water diffusivity and polymer chain mobility, which reduce the resistance to water penetration into the network. However, stronger swelling at elevated temperature does not necessarily indicate better plugging stability. In low-crosslinking-density particles, excessive thermal swelling may soften the network and weaken structural retention, whereas highly crosslinked particles remain more constrained but less deformable.

The effect of salinity on swelling behavior was opposite to that of temperature. As shown in Figure 4c, the equilibrium swelling ratios of all samples decreased as the salinity of simulated formation water increased from 50,000 to 150,000 mg/L. For EGP-2, the equilibrium swelling ratio decreased from approximately 8.1 g/g at 50,000 mg/L to approximately 6.2 g/g at 150,000 mg/L. This decrease is mainly associated with ionic screening and reduced osmotic driving force under high-salinity conditions. Na^+^, Ca^2+^, and Mg^2+^ compress the hydration layer around hydrophilic groups and weaken water uptake into the polymer network. Although AMPS-derived sulfonic groups improve salt-tolerant hydration, they cannot completely eliminate the swelling suppression caused by high salinity.

Overall, the swelling behavior reflects a coupled response among the PEGDA-imposed network constraint, temperature-enhanced water diffusion, and salinity-induced ionic screening. EGP-1 swelled strongly but tended toward structural relaxation, whereas EGP-3 and EGP-C were more constrained and less deformable. EGP-2 provided a more balanced hydrated state, with sufficient water uptake for wall contact and elastic deformation while avoiding excessive swelling-induced size broadening. This balance is critical for subsequent particle bridging, accumulation, and retention in fracture channels.

### 2.3. Rheological and Compression Stability of Hydrated Gel Particles

**Figure 5:**
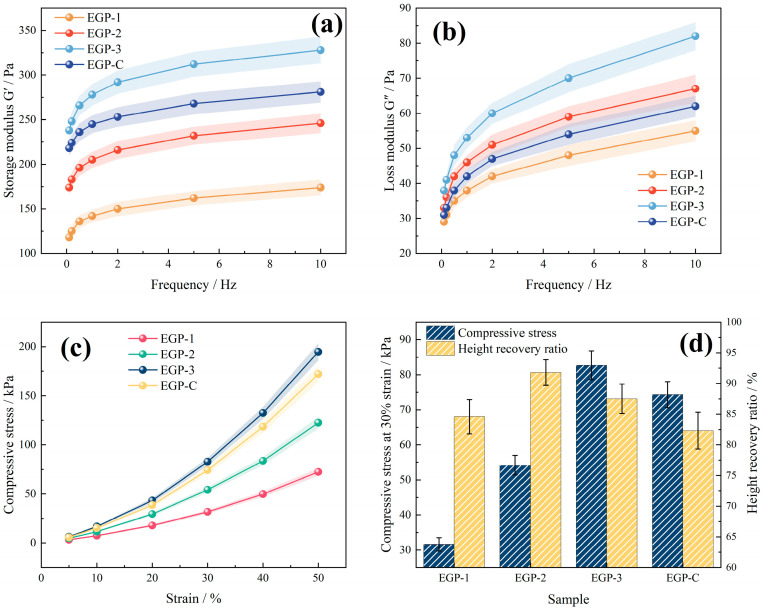
Rheological and compression stability of hydrated gel particles. (**a**) Storage modulus G′ as a function of frequency; (**b**) loss modulus G″ as a function of frequency; (**c**) compressive stress–strain curves; (**d**) comparison of compressive stress at 30% strain and height recovery ratio. Error bars represent standard deviations, *n* = 3.

According to the rheological and compression testing methods described in Section 4.4, the viscoelasticity, compressive resistance, and elastic recovery of hydrated EGP-1, EGP-2, EGP-3, and EGP-C gel particles were evaluated to determine their ability to resist fluid erosion, undergo elastic deformation, and maintain bridging-packing structures during fracture temporary plugging. Figure 5 summarizes the viscoelastic response, compressive stress–strain behavior, compressive stress at 30% strain, and post-compression height recovery of the hydrated particles. These parameters were used to evaluate whether the particles could provide sufficient mechanical support without losing the deformability required for fracture adaptation.

As shown in Figure 5a,b, all hydrated gel particle suspensions exhibited G′ values higher than G″ over the frequency range of 0.1–10 Hz, indicating that the elastic response dominated over viscous flow. At 1 Hz, the G′ values of EGP-1, EGP-2, EGP-3, and EGP-C were 142, 205, 278, and 245 Pa, respectively, while the corresponding G″ values were 38, 46, 53, and 42 Pa. The increase in G′ from EGP-1 to EGP-3 reflects the enhanced elastic support caused by higher PEGDA crosslinking density. However, the rheological data should not be interpreted simply as “higher G′ is better”. For fracture plugging, excessive rigidity can reduce particle deformation and fracture-wall adaptability, whereas insufficient elasticity can lead to particle collapse or erosion under flow.

This mechanical balance explains the different roles of the four formulations. EGP-1 had the lowest G′, consistent with its loose and highly swollen network, but its weak elastic support may reduce packing stability under displacement. EGP-3 and EGP-C showed higher G′ values, indicating stronger network rigidity; however, this rigidity may limit reversible deformation in confined fracture spaces. EGP-2 maintained an intermediate G′ and a clear predominance of G′ over G″, suggesting that it combined elastic support with limited viscous dissipation. This viscoelastic state is more favorable for particles that must deform during fracture entry and then sustain contact forces after bridging.

Compression testing further reflected the load-bearing behavior of hydrated gel particles under confined conditions. As shown in Figure 5c, the compressive stress increased nonlinearly with strain for all samples, reflecting particle rearrangement, pore compression, and progressive network loading. At 30% strain, EGP-1, EGP-2, EGP-3, and EGP-C showed compressive stresses of 31.6, 54.2, 82.7, and 74.3 kPa, respectively. The higher values of EGP-3 and EGP-C indicate stronger load-bearing capacity, but they also correspond to more constrained networks. In contrast, EGP-2 provided sufficient compressive resistance while avoiding the excessive stiffening observed for EGP-3 and EGP-C.

The height recovery ratio further evaluates the structural recovery of particles after compression-induced disturbance. As shown in Figure 5d, EGP-2 showed the highest height recovery ratio of 91.8%, exceeding those of EGP-1, EGP-3, and EGP-C. The recovery ratio of EGP-2 was significantly higher than those of the other formulations (*p* < 0.05), supporting its superior reversible deformation capacity after compression. EGP-1 was more deformable but lacked sufficient skeleton support, whereas EGP-3 and EGP-C were stronger but less recoverable because of their denser or more rigid crosslinked networks. Therefore, EGP-2 achieved a more suitable balance between compressive strength and elastic recovery.

For fracture plugging, hydrated particles must pass through the injection path, deform at local constrictions, form bridge-packing structures, and resist erosion after retention. The rheological and compression results show that EGP-2 best satisfies this mechanical window: it is stronger than the highly swollen EGP-1, more deformable and recoverable than the rigid EGP-3 and EGP-C, and better suited for stable but reversible fracture plugging. This mechanical balance provides the basis for the single-fracture and multi-fracture flow experiments discussed below.

### 2.4. Ester-Bond-Cleavage-Induced Self-Degradation Behavior

**Figure 6:**
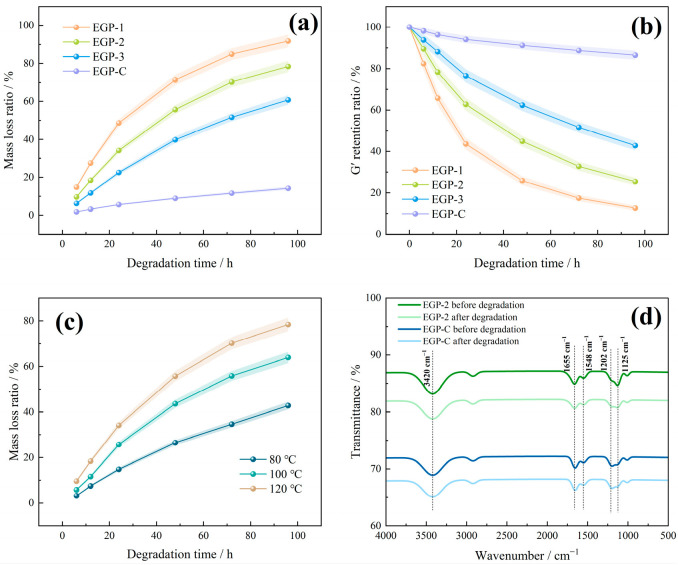
Ester-bond-cleavage-induced self-degradation behavior of gel particles. (**a**) Mass loss ratio as a function of degradation time at 120 °C and 100,000 mg/L salinity; (**b**) G′ retention ratio after sealed aging at 120 °C and 100,000 mg/L salinity; (**c**) effect of temperature on the mass loss ratio of EGP-2; (**d**) FTIR spectral changes in EGP-2 and EGP-C before and after degradation. Error bars represent standard deviations, *n* = 3.

According to the self-degradation test described in Section 4.4, the structural attenuation of EGP-1, EGP-2, EGP-3, and EGP-C in high-temperature simulated formation water was evaluated. The self-degradation process was characterized by mass loss ratio, G′ retention ratio measured after sealed thermal aging, temperature-dependent degradation response, and FTIR spectral changes before and after degradation. Figure 6 summarizes the time-dependent mass loss, G′ retention after sealed aging, temperature-dependent degradation of EGP-2, and FTIR changes before and after aging. These results were used to determine whether the PEGDA-derived ester structures could induce gradual network cleavage in a high-temperature aqueous environment.

As shown in Figure 6a, the mass loss ratios of the EGP samples continuously increased with degradation time, while the degradation rates differed clearly with PEGDA crosslinking density. EGP-1 reached a mass loss ratio of 48.6% at 24 h and further increased to 91.8% after 96 h, reflecting rapid structural relaxation and particle disintegration in the high-temperature aqueous environment. The mass loss ratio of EGP-2 increased steadily and reached 78.4% after 96 h, showing substantial but delayed network attenuation. In contrast, EGP-3 showed a mass loss ratio of 60.7% after 96 h, which was lower than those of EGP-1 and EGP-2, indicating that stronger PEGDA-imposed network constraints slowed hydrolytic degradation. EGP-C maintained a much lower mass loss ratio, reaching only 14.2% after 96 h, indicating limited aging or surface erosion rather than pronounced bulk network disintegration.

The variation in G′ retention ratio measured after sealed thermal aging further reflected the attenuation of mechanical support within the crosslinked network during degradation. As shown in Figure 6b, the G′ retention ratios of the EGP samples after different sealed aging times continuously decreased with degradation time, following a trend consistent with the mass loss results. After 96 h, the G′ retention ratios of EGP-1, EGP-2, and EGP-3 decreased to 12.6%, 25.4%, and 42.8%, respectively, whereas EGP-C retained 86.5% of its initial G′. The coupled increase in mass loss and decrease in G′ retention indicates that degradation of the EGP samples was not limited to surface erosion, but involved progressive loss of crosslinked network integrity. It should be noted that these G′ retention values were obtained after sealed thermal aging followed by rheological measurement under the non-boiling conditions described in Section 4.4.3, rather than by direct open-system measurement at 120 °C.

Temperature markedly promoted the self-degradation process of EGP-2. As shown in Figure 6c, the mass loss ratio of EGP-2 increased significantly as the temperature increased from 80 to 120 °C. At 72 h, the mass loss ratios of EGP-2 at 80, 100, and 120 °C were 34.6%, 55.8%, and 70.2%, respectively; after 96 h, the corresponding values further increased to 42.8%, 63.9%, and 78.4%. This temperature-dependent acceleration can be attributed to enhanced water diffusion, increased chain mobility, and faster cleavage of hydrolysable crosslinking sites. For temporary plugging, this response is important because the particles should remain stable during placement and early diversion, but gradually lose network integrity under reservoir temperature to reduce long-term residue retention.

The FTIR comparison in Figure 6d was used as supporting evidence for chemical changes during degradation, rather than as an independent proof of ester-bond cleavage. Consistent with the structural interpretation of PEGDA incorporation in Section 2.1.1, the C–O–C absorption near 1125 cm^−1^ became weaker after EGP-2 degradation, which is consistent with the attenuation of PEGDA-related crosslinking structures in the high-temperature aqueous environment. In contrast, EGP-C showed only minor spectral changes under the same aging condition, supporting the relative stability of the MBAA-crosslinked network during high-temperature exposure. Therefore, the FTIR results were interpreted together with the mass loss and G′ retention data, rather than being used alone to determine the degradation mechanism.

The degradation behavior of the EGP series is better interpreted as a progressive loss of network integrity rather than simple surface erosion. EGP-1 degraded too rapidly to provide durable structural retention, whereas EGP-3 retained more network integrity and may have left more residues. EGP-2 retained enough structure during the plugging period but still underwent substantial attenuation during high-temperature aging, which is the desired response for temporary and recoverable fracture plugging. Thus, EGP-2 represents a balanced degradation window, avoiding premature collapse before effective plugging while enabling delayed network weakening for subsequent deplugging and flowback recovery.

### 2.5. Temporary Plugging Behavior in Single-Fracture Systems

**Figure 7:**
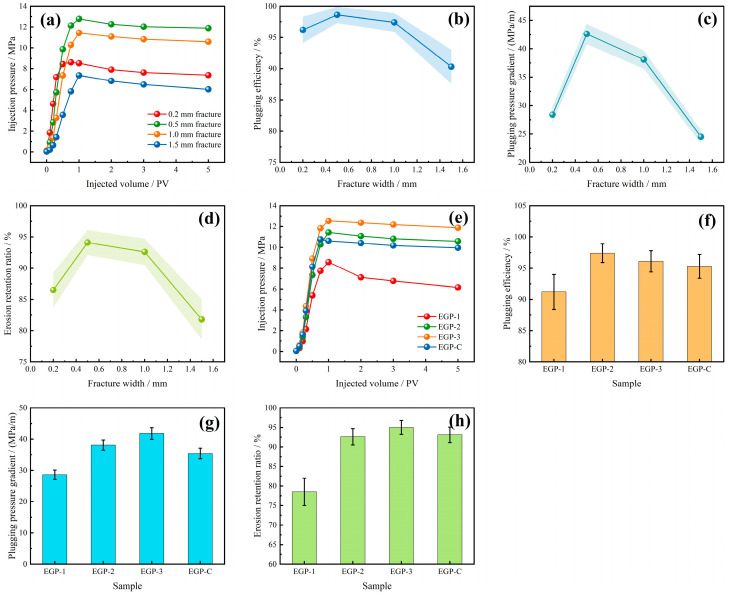
Temporary plugging behavior of gel particles in single-fracture systems. (**a**) Representative pressure response curves of EGP-2 during injection under different fracture widths; (**b**) plugging efficiency of EGP-2 under different fracture widths; (**c**) plugging pressure gradient of EGP-2 under different fracture widths; (**d**) erosion retention ratio of EGP-2 under different fracture widths; (**e**) representative pressure response curves of different samples in a 1.0 mm fracture; (**f**) plugging efficiency of different samples in a 1.0 mm fracture; (**g**) plugging pressure gradient of different samples in a 1.0 mm fracture; (**h**) erosion retention ratio of different samples in a 1.0 mm fracture. Error bars represent standard deviations, *n* = 3.

According to the single-fracture plugging test described in Section 4.4, the plugging ability of hydrated gel particles in fractures with different apertures was evaluated. Figure 7 summarizes the pressure response, plugging efficiency, plugging pressure gradient, and erosion retention of EGP-2 under different fracture widths, and compares the plugging behavior of different formulations in a 1.0 mm fracture. These results were used to determine whether hydrated particles could effectively migrate, bridge, accumulate, and compact within fracture channels, providing a basis for subsequent multi-fracture selective plugging experiments.

As shown in Figure 7a, during the injection of the particle suspension, the inlet pressure of the fracture models underwent a transition from a low-pressure migration stage to a rapid pressure-build-up stage. In the initial stage, pressure increased slowly, indicating that hydrated particles could enter the fracture channels with the injected fluid. As particles gradually retained within the fractures, pressure increased rapidly, corresponding to the formation of particle bridging, accumulation, and local compaction structures. For EGP-2, the 0.5 and 1.0 mm fractures showed more stable pressure build-up and pressure retention than the 0.2 and 1.5 mm fractures, suggesting that these apertures were more suitable for effective internal bridging rather than only inlet plugging or weak multi-particle accumulation.

As shown in Figure 7b–d, the plugging performance of EGP-2 differed clearly among fracture widths. EGP-2 achieved plugging efficiencies of 98.6% and 97.4% in the 0.5 and 1.0 mm fractures, respectively, with corresponding plugging pressure gradients of 42.6 and 38.1 MPa/m and erosion retention ratios of 94.1% and 92.6%. These results indicate that the 0.5–1.0 mm aperture range provided a more favorable particle–fracture size match. In this range, hydrated EGP-2 particles could enter the fracture, deform under confinement, bridge with neighboring particles, and form a compacted plugging zone. In contrast, the 0.2 mm fracture showed a high plugging efficiency of 96.2%, but its plugging pressure gradient and erosion retention ratio were only 28.4 MPa/m and 86.5%, respectively. For the 1.5 mm fracture, the plugging efficiency, plugging pressure gradient, and erosion retention ratio decreased to 90.3%, 24.5 MPa/m, and 81.8%, respectively. Thus, high plugging efficiency alone did not necessarily indicate stable fracture plugging. The pressure gradient and erosion retention ratio were more useful for distinguishing internal bridge-packing from near-inlet blockage or weak accumulation in oversized fractures.

As shown in Figure 7e–h, the four formulations showed different plugging responses in the 1.0 mm fracture. EGP-1 generated a lower pressure build-up and weaker pressure retention, indicating insufficient structural support under continuous displacement. EGP-3 showed the highest plugging pressure gradient of 41.8 MPa/m, and EGP-C also exhibited strong erosion resistance; however, their higher rigidity or nonhydrolysable network structure may reduce fracture adaptability and increase the risk of difficult deplugging after treatment. EGP-2 achieved a plugging efficiency of 97.4%, a plugging pressure gradient of 38.1 MPa/m, and an erosion retention ratio of 92.6%. Although its pressure gradient was not the highest, EGP-2 provided a more suitable temporary plugging response because it balanced particle deformability, bridge-packing stability, erosion resistance, and subsequent degradability.

Therefore, the single-fracture results connect the material properties established in Section 2.1, Section 2.2 and Section 2.3 with fracture-scale plugging behavior. EGP-2 was not selected because it produced the maximum pressure gradient, but because it formed stable plugging structures in the most suitable aperture range while retaining the degradable network required for later deplugging and flowback recovery. This behavior provides the basis for evaluating whether the same particles can redistribute flow in a multi-fracture system.

### 2.6. Selective Plugging and Flow Diversion in Multi-Fracture Systems

**Figure 8:**
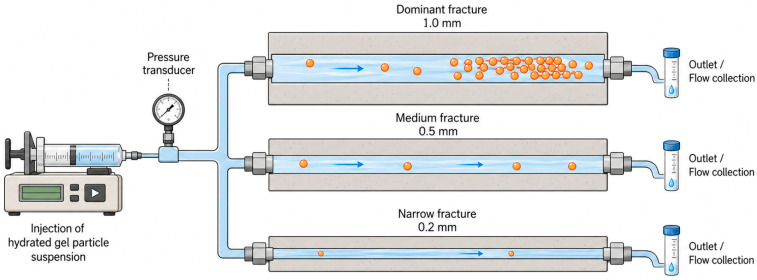
Schematic illustration of the three-parallel-fracture model.

**Figure 9:**
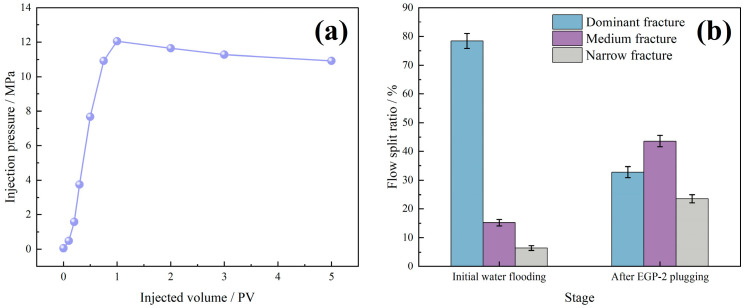
Selective plugging and flow diversion behavior of EGP-2 in the multi-fracture system. (**a**) Representative pressure response curve of EGP-2 in the multi-fracture model; (**b**) changes in flow split ratios of different fracture channels before and after EGP-2 plugging. Error bars represent standard deviations, *n* = 3. For Figure 9b, statistical comparisons were performed among fracture channels before and after plugging, and *p* < 0.05 was considered statistically significant.

According to the multi-fracture parallel model test described in Section 4.4, the selective plugging and flow diversion abilities of gel particles in a heterogeneous multi-fracture system were further evaluated. Figure 8 shows the schematic illustration of the three-parallel-fracture model, in which fracture widths of 1.0, 0.5, and 0.2 mm were used to represent the dominant, medium, and narrow fractures, respectively. Figure 9 and Table 2 and Table 3 were used to evaluate whether particle retention in the dominant fracture could be translated into effective flow redistribution rather than simple local blockage.

During the initial water flooding stage, the multi-fracture model exhibited a pronounced preferential flow channel effect. As shown in Figure 9b, before gel particle injection, the flow split ratio of the 1.0 mm dominant fracture reached 78.4%, whereas those of the 0.5 and 0.2 mm fractures were only 15.2% and 6.4%, respectively. This flow distribution verifies the function of the model in Figure 8: the dominant fracture controlled most of the injected fluid, while the medium and narrow fractures remained poorly swept. Therefore, the key question was not whether the particles could generate pressure, but whether they could reduce the hydraulic dominance of the 1.0 mm fracture and redirect flow into the smaller fractures.

After EGP-2 injection, the inlet pressure increased to approximately 12.05 MPa at 0.5–1.0 PV and remained within 10.92–11.64 MPa during subsequent water flooding. Rather than describing only a pressure-rise process, this response indicates that EGP-2 formed a retained bridge-packing structure with sufficient erosion resistance in the dominant fracture. After plugging, the flow split ratio of the dominant fracture decreased from 78.4% to 32.8%, whereas those of the medium and narrow fractures increased to 43.6% and 23.6%, respectively. The combined flow split ratio of the medium and narrow fractures therefore increased from 21.6% to 67.2%, corresponding to a flow diversion efficiency of 58.2% in Table 2. These data demonstrate that EGP-2 converted a dominant-channel flow pattern into a redistributed flow pattern, which is the essential criterion for multi-fracture diversion.

Different crosslinking produced different redistribution efficiencies in the multi-fracture system. As shown in Table 3, EGP-1 showed a lower flow diversion efficiency of 42.2%, indicating that strong swelling alone was insufficient when skeleton support was too weak to maintain resistance in the dominant fracture. EGP-3 and EGP-C achieved higher diversion efficiencies of 50.8% and 53.6%, respectively, but their stronger network rigidity or nonhydrolysable crosslinking structure may reduce deformation adaptability or increase the risk of residue retention after treatment. In comparison, EGP-2 achieved the highest diversion efficiency of 58.2% and the largest reduction in dominant-fracture flow split. This result is consistent with the preceding particle-size, swelling, mechanical, degradation, and single-fracture plugging results: EGP-2 had sufficient hydrated size for bridge formation, enough elasticity for retention, and an ester-cleavable network for subsequent recovery.

Thus, the multi-fracture results show that EGP-2 acted as a temporary flow-diversion agent rather than a simple plugging material. Its advantage lies in increasing resistance selectively in the dominant fracture while restoring flow contribution from medium and narrow fractures, without sacrificing the degradable structure required for later deplugging and flowback.

### 2.7. Controlled Deplugging and Flowback Recovery

**Figure 10:**
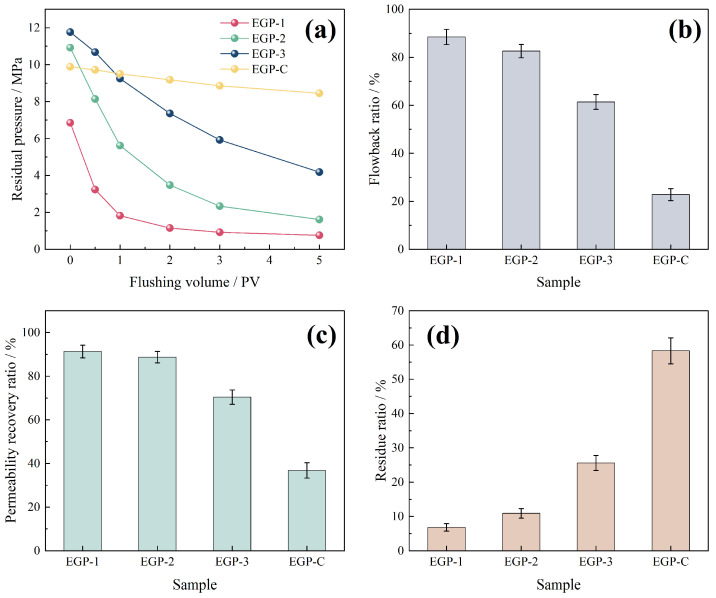
Controlled deplugging and flowback recovery behavior of gel particles. (**a**) Representative pressure variation curves of different samples during flowback after aging at 120 °C for 72 h; (**b**) flowback ratio of different samples; (**c**) permeability recovery ratio of different samples; (**d**) residue ratio of different samples. Error bars represent standard deviations, *n* = 3.

According to the controlled deplugging and flowback recovery test described in Section 4.4, the deplugging pressure response, flowback capacity, permeability recovery, and residue characteristics of different gel particles were evaluated after single-fracture plugging. A 1.0 mm fracture model was selected as the representative evaluation object because the previous results showed that particles could enter this fracture aperture and form relatively stable bridging-packing structures. Figure 10 summarizes the pressure response during flowback, particle flowback ratio, permeability recovery ratio, and residue ratio after aging at 120 °C for 72 h. These indicators were used to evaluate whether the plugging structure could be removed after service without leaving severe residual blockage.

As shown in Figure 10a, different samples exhibited distinct deplugging pressure responses after high-temperature aging. EGP-1 showed a rapid pressure decline, indicating easy removal but also reflecting the weak plugging stability discussed in Section 2.5. EGP-3 and EGP-C showed much slower pressure decreases, and EGP-C maintained a high residual pressure during flowback, indicating that the nonhydrolysable MBAA-crosslinked structure could not undergo effective network attenuation in hot water. In contrast, EGP-2 exhibited a gradual pressure decrease followed by stabilization, suggesting controlled deplugging rather than premature collapse or persistent residual blockage.

As shown in Figure 10b, the flowback ratio directly reflected the discharge capacity of degraded particles and residues. EGP-1 exhibited the highest flowback ratio of 88.5%, consistent with its rapid network attenuation. However, this high flowback ratio should not be interpreted independently from plugging stability, because EGP-1 had already shown weaker bridge-packing resistance in the previous plugging tests. EGP-2 achieved a flowback ratio of 82.6%, slightly lower than that of EGP-1 but much higher than those of EGP-3 and EGP-C. This result indicates that EGP-2 underwent sufficient network softening after aging to allow particle removal, while still retaining stronger plugging performance before deplugging. EGP-3 and EGP-C showed lower flowback ratios of 61.4% and 22.8%, respectively, reflecting stronger residual retention caused by higher crosslinking density or a nonhydrolysable network.

The permeability recovery ratio is an important indicator for evaluating whether a temporary plugging material causes long-term fracture damage. Figure 10c shows that EGP-1 and EGP-2 both achieved high permeability recovery ratios of 91.3% and 88.7%, respectively. Although EGP-1 showed a slightly higher recovery, this recovery was mainly associated with rapid structural disruption rather than a balanced plugging–deplugging response. EGP-2 achieved nearly 90% permeability recovery while also showing stable plugging and diversion behavior in Section 2.5 and Section 2.6. Therefore, EGP-2 provided a more meaningful recovery performance because conductivity restoration was achieved without sacrificing the temporary plugging function. In contrast, EGP-3 and EGP-C showed lower permeability recovery ratios of 70.4% and 36.8%, respectively, indicating that insufficient degradation left residual flow resistance in the fracture.

As shown in Figure 10d, the residue ratio provides a direct measure of post-treatment retention. EGP-C showed the highest residue ratio of 58.3%, indicating that a large number of nondegradable particles remained in the fracture after aging and flowback. EGP-3 showed a residue ratio of 25.6%, suggesting that excessive crosslinking delayed network attenuation and increased residual retention. EGP-1 showed the lowest residue ratio of 6.8%, but this low residue was accompanied by insufficient plugging stability. EGP-2 showed a low residue ratio of 10.9%, which confirms that its degradation was sufficient to reduce persistent fracture blockage while avoiding the premature loss of plugging integrity observed for EGP-1. The differences in flowback ratio, permeability recovery ratio, and residue ratio among the formulations were statistically significant (*p* < 0.05), further supporting the distinct recovery behavior of the PEGDA-crosslinked and MBAA-crosslinked systems.

Taken together, Figure 10a–d shows that EGP-2 achieved a controlled plugging–deplugging transition. The pressure response confirmed gradual deplugging, the flowback ratio confirmed effective particle removal, the permeability recovery ratio confirmed restored fracture conductivity, and the residue ratio confirmed limited post-treatment retention. This behavior results from the coupling between PEGDA-related network attenuation and hydrodynamic flowback removal, allowing EGP-2 to provide stable temporary plugging during treatment and low-residue recovery after aging.

To further place the performance of EGP-2 in the context of reported temporary plugging materials, key parameters of representative degradable gel particle systems and temporary plugging agents were compared with those of the present system, as summarized in Table 4. Previous degradable preformed particle gels have shown strong temporary plugging ability and good permeability recovery; for example, Zhu et al. [12] reported a DPPG system with a temporary plugging efficiency above 94% and permeability recovery above 90%. Zhang et al. [14] further showed that PEGDA-related degradable particle gels could reach swelling ratios from 7 to 33 times and complete degradation within 40–210 min, depending on the molecular weight of the crosslinking agent. These studies demonstrate the feasibility of degradable particle gels for temporary plugging, but most evaluations mainly focused on swelling, degradation time, plugging pressure, or core-scale recovery. In comparison, EGP-2 showed a balanced swelling ratio of 7.12 g/g, plugging efficiencies of 98.6% and 97.4% in 0.5 and 1.0 mm fractures, respectively, a mass loss ratio of 78.4% after 96 h at 120 °C, a flowback ratio of 82.6%, a permeability recovery ratio of 88.7%, and a residue ratio of 10.9%. More importantly, EGP-2 also achieved a flow diversion efficiency of 58.2% in the three-parallel-fracture model, which directly reflects flow redistribution under competitive fracture conductivities. Therefore, the advantage of EGP-2 is not a single maximum value in swelling, plugging, or degradation, but the integration of stable temporary plugging, multi-fracture diversion, controlled degradation, and low-residue recovery within one particle system.

### 2.8. Plugging–Diversion–Deplugging Mechanism in Multi-Fracture Systems

**Figure 11:**
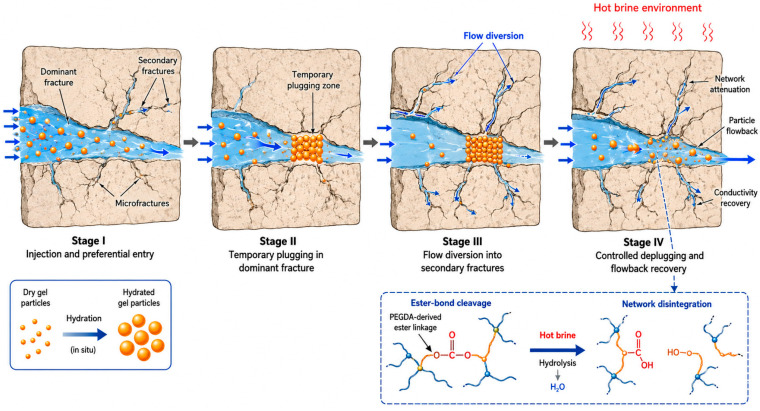
Schematic illustration of the plugging–diversion–deplugging mechanism of ester-bond-cleavable self-degradable gel particles in the multi-fracture system, highlighting in situ hydration, temporary plugging by hydrated particle packing, and subsequent attenuation of the hydrated particle pack under hot-brine conditions.

The preceding results support a sequential plugging–diversion–deplugging mechanism for ester-bond-cleavable gel particles in heterogeneous fracture networks. This mechanism links molecular design to fracture-scale behavior: PEGDA introduces hydrolysable crosslinking points, the hydrated particles acquire deformable bridging dimensions, the particle pack temporarily increases resistance in dominant fractures, and high-temperature aging subsequently weakens the network to enable flowback recovery. Figure 11 summarizes this structure–function relationship, showing how ester-bond-cleavable particles integrate preferential entry, bridge-packing, flow redistribution, attenuation of the hydrated particle pack, and low-residue recovery within one temporary plugging system. It should be emphasized that Figure 11 does not represent direct breakdown of the dry particles. Instead, the schematic describes in situ hydration of the injected dry particles, temporary plugging by the resulting hydrated particles, and subsequent attenuation of the hydrated gel-particle packing structure under hot-brine conditions.

At the injection and plugging stage, the lower flow resistance of the 1.0 mm dominant fracture promotes preferential particle entry. For EGP-2, the hydrated particle size, moderate elasticity, and compression recovery provide a suitable mechanical window for fracture entry, deformation, bridging, and retention. The injected particles initially enter the fracture in the dry state, but the plugging body shown in Figure 11 is formed after in situ hydration, swelling, and compaction of these particles within the dominant fracture. Particles that are insufficiently supported tend to lose plugging stability, whereas overly rigid or nondegradable particles may reduce fracture adaptability or leave residues after treatment. Thus, the key factor is not maximum swelling or maximum rigidity, but the balance between deformability and skeleton support.

After bridge-packing is established in the dominant fracture, the flow resistance among parallel fractures is redistributed. In the multi-fracture experiment, the dominant-fracture flow split decreased from 78.4% to 32.8%, while the combined flow split of the medium and narrow fractures increased from 21.6% to 67.2%. These changes indicate that EGP-2 converted preferential flow through the dominant fracture into controlled flow redistribution across the fracture network. Therefore, its role is better defined as temporary flow diversion rather than simple local blockage.

At the deplugging stage, the PEGDA-related ester-containing crosslinks gradually attenuate under high-temperature aqueous conditions. This network weakening softens the compacted particle pack, reconnects internal flow pathways, and allows degraded residues to be removed during flowback. Therefore, the deplugging process shown in Figure 11 should be understood as attenuation and disintegration of the hydrated particle-packing structure, rather than direct fragmentation of the initially injected dry particles. The flowback ratio of 82.6%, permeability recovery ratio of 88.7%, and residue ratio of 10.9% confirm that EGP-2 retained sufficient plugging integrity during treatment while avoiding persistent fracture blockage after aging. Accordingly, deplugging is not merely physical washout, but a coupled process involving ester-bond-related network attenuation and hydrodynamic removal.

Overall, the plugging–diversion–deplugging behavior of EGP-2 arises from the coupling of PEGDA-regulated network structure, hydration-induced fracture matching, mechanical bridge stability, and thermally assisted network attenuation. This mechanism provides a design basis for temporary plugging materials that must achieve effective flow diversion during treatment and conductivity recovery after treatment in heterogeneous multi-fracture reservoirs. Compared with previously reported PEGDA-based degradable gels that mainly emphasize hydrolysable network formation or bulk degradation behavior, the present system highlights the coupling between ester-bond-cleavable crosslinking, particle-scale fracture matching, selective flow redistribution, and low-residue deplugging in a multi-fracture environment. In this sense, the superiority of the present concept lies not in simple bulk-gel degradation, but in the use of dry injectable particles that hydrate in situ, form a temporary plugging body under fracture-flow competition, and then undergo delayed attenuation of the hydrated particle pack to achieve both diversion and conductivity recovery.

## 3. Conclusions

In this study, ester-bond-cleavable self-degradable gel particles were designed to address the difficulty of balancing temporary plugging strength and post-treatment removability in multi-fracture reservoir systems. By incorporating PEGDA as a hydrolysable crosslinker into an AAm/AMPS/NVP polymer framework, gel particles with tunable swelling, mechanical stability, and degradation behavior were obtained. Structural characterization confirmed the formation of PEGDA-related crosslinked networks, while the comparative MBAA-crosslinked control demonstrated the importance of degradable crosslinking chemistry in enabling later-stage network attenuation. Among the prepared samples, EGP-2 showed the most balanced performance, combining moderate hydration swelling, sufficient elastic support, and favorable compression recovery.

The single-fracture and multi-fracture experiments demonstrated that EGP-2 could form stable bridging and packing structures in millimeter-scale fractures and effectively regulate flow distribution under competitive flow conditions. In the three-parallel-fracture model, the dominant-fracture flow split ratio decreased from 78.4% to 32.8%, while the combined flow split ratio of the medium and narrow fractures increased to 67.2%, confirming that the particles promoted flow diversion rather than merely increasing local flow resistance. The degradation and flowback results further showed that the ester-bond-cleavable network enabled controlled structural attenuation under high-temperature aqueous conditions, allowing the plugged particles to soften, fragment, and be removed during subsequent flushing. EGP-2 achieved a permeability recovery ratio of 88.7% and a residue ratio of 10.9%, indicating a favorable balance between plugging retention and low-residue recovery.

These results confirm that ester-bond-cleavable gel particles provide an effective strategy for integrating temporary plugging, flow diversion, and controlled deplugging within a single material system. This work offers a structural design route for low-residue conformance control materials in fractured reservoirs. Unlike conventional degradable gel particles or PEGDA-based gels evaluated mainly by plugging strength or degradation behavior alone, this study emphasizes a complete performance chain from fracture-scale selective plugging to flow redistribution and low-residue recovery. Further work should focus on long-term stability under complex formation-water compositions, cyclic plugging–deplugging performance, and validation in more realistic core-scale fracture networks.

## 4. Materials and Methods

### 4.1. Experimental Materials

The materials used in this study are summarized in Table 5.

### 4.2. Experimental Instruments

The major experimental instruments and testing systems are summarized in Table 6.

### 4.3. Synthesis Procedure

**Figure 12:**
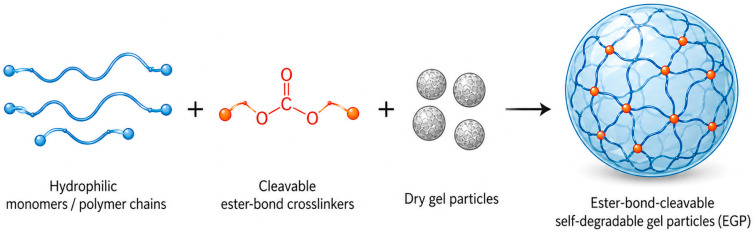
Schematic illustration of the composition and structural design of ester-bond-cleavable self-degradable gel particles.

To prepare self-degradable gel particles with both fracture plugging and controlled deplugging capabilities, an aqueous free-radical crosslinking method was used to construct an ester-bond-cleavable gel network. Acrylamide (AAm), 2-acrylamido-2-methylpropanesulfonic acid (AMPS), and N-vinyl-2-pyrrolidone (NVP) were used as the main monomer, salt-tolerant functional monomer, and thermally stabilizing auxiliary monomer, respectively. Poly(ethylene glycol) diacrylate (PEGDA, Mn = 575 g/mol) was used as a hydrolysable ester-bond-containing crosslinker, while N,N′-methylenebisacrylamide (MBAA) was used to prepare the nondegradable control sample. The composition and network design of the system are illustrated in Figure 12.

Specifically, 80.0 mL of deionized water was added to a 250 mL glass beaker and stirred at 25 °C and 300 rpm for 5 min. Subsequently, AAm (9.00 g), AMPS (3.75 g), and NVP (2.25 g) were sequentially added, giving a total monomer mass of 15.00 g. The mixture was stirred at 400 rpm for 20 min until a transparent and homogeneous solution was obtained. The pH of the solution was adjusted to 7.0–7.2 using 1.0 mol/L NaOH solution to ensure the stability of the subsequent free-radical polymerization process.

PEGDA was then added to the monomer solution at dosages of 0.15, 0.30, and 0.45 g, corresponding to 1.0, 2.0, and 3.0 wt% of the total monomer mass, respectively. The resulting samples were denoted as EGP-1, EGP-2, and EGP-3. To establish a nondegradable control group, EGP-C was prepared by replacing PEGDA with 0.045 g of MBAA. The formulations of all samples are listed in Table 7. After the addition of the crosslinker, the solution was further stirred at 300 rpm for 15 min, followed by nitrogen purging for 20 min to remove dissolved oxygen. After deoxygenation, ammonium persulfate (APS, 0.10 g) and sodium bisulfite (NaHSO_3_, 0.10 g) were added as the redox initiation system. The mixture was rapidly stirred for 2 min after initiator addition and then transferred into sealed glass molds. Polymerization was carried out in a thermostatic water bath at 45 °C for 3 h, followed by aging at room temperature for 12 h.

After polymerization, the bulk gel was removed from the mold and cut into small cubes of approximately 5 mm × 5 mm × 5 mm. The gel pieces were washed with deionized water for 24 h, during which the washing water was replaced every 6 h to remove residual monomers and soluble components. The washed gels were dried at 45 °C to constant weight, mechanically crushed, and then sieved to obtain gel particles with three particle-size fractions: 50–300 μm, 300–600 μm, and 600–1000 μm. Unless otherwise specified, the 300–600 μm fraction was used in subsequent experiments to ensure an appropriate particle–fracture matching relationship with the millimeter-scale fracture models. The dry gel particles were stored in sealed polyethylene bottles and kept in a room-temperature desiccator before use.

The synthesis yield was calculated to clarify the reproducibility of the particle preparation process. The dry-gel yield was defined as the mass of dried gel after washing and drying divided by the total solid feed mass, including monomers, crosslinkers, and initiators. The target particle-fraction yield was defined as the mass of 300–600 μm particles obtained after crushing and sieving divided by the dried gel mass. The results are summarized in Table 8. The dry-gel yields of all samples were within a similar range of 88.7–90.8%, indicating that the selected polymerization conditions produced stable bulk gels for both PEGDA-crosslinked and MBAA-crosslinked systems. The target particle-fraction yield was more sensitive to crosslinking structure. EGP-1 produced more fine fragments because of its lower crosslinking density, whereas EGP-3 and EGP-C showed higher particle recovery due to their denser networks. EGP-2 maintained a moderate and stable target particle-fraction yield, indicating that an intermediate PEGDA crosslinking density helped balance particle integrity and fragmentation behavior during crushing and sieving.

EGP-C was used as the nondegradable control sample to distinguish PEGDA ester-bond-hydrolysis-induced network attenuation from structural changes caused by ordinary thermal aging, swelling variation, or mechanical erosion. The subsequent swelling, mechanical, degradation, and plugging experiments were all performed based on the above sample system.

### 4.4. Experimental Method

#### 4.4.1. Particle Size Distribution Measurement

The particle size distribution of the ester-bond-cleavable self-degradable gel particles was determined using microscopic image analysis. Dry EGP-1, EGP-2, EGP-3, and EGP-C particles obtained after standard sieving were evenly spread on clean glass slides and imaged using an optical microscope. To minimize the influence of particle overlap and compression-induced deformation, the samples were not pressed before observation and were only gently shaken to achieve natural dispersion. For each sample, at least 100 particles were randomly selected for equivalent diameter analysis, and D10, D50, and D90 were calculated. D50 was used as the median particle size for subsequent particle–fracture size matching analysis.

To evaluate the particle size change after hydration, 0.10 g of dry gel particles was dispersed in 100 mL of simulated formation water and hydrated at 80 °C for 12 h. After hydration, the particles were separated using a 100-mesh sieve, and the surface free water was gently removed with filter paper. The hydrated particles were then placed on glass slides, imaged using an optical microscope, and the equivalent diameters of at least 100 particles were measured. All particle size measurements were performed in triplicate, and the results are expressed as mean ± standard deviation.

#### 4.4.2. Hydration Swelling Test

The hydration swelling behavior of gel particles in simulated formation water was evaluated using a gravimetric method. Specifically, 0.10 g of dry gel particles was added to 100 mL of simulated formation water and hydrated for different durations at preset temperatures. The test temperatures were set at 60, 80, 100, and 120 °C, and the hydration times were 0.5, 1, 2, 4, 8, 12, and 24 h. To assess the effect of salinity on particle swelling, the salinities of the simulated formation water were set at 50,000, 100,000, and 150,000 mg/L. After reaching the target hydration time, the hydrated particles were separated using a 100-mesh sieve, and the surface free water was gently removed with filter paper. The hydrated particles were then weighed immediately.

The mass swelling ratio of gel particles was calculated using Equation (1):

(1) $$S_{w}=\frac{m_{t}-m_{0}}{m_{0}}$$

where $S_{w}$ is the mass swelling ratio; $m_{0}$ is the initial mass of dry gel particles, g; and $m_{t}$ is the mass of hydrated gel particles after hydration for time *t*, g. Each experiment was repeated three times, and the results are expressed as mean ± standard deviation.

#### 4.4.3. Rheological Characterization

The viscoelastic properties of hydrated gel particle suspensions were characterized using a rotational rheometer. Dry gel particles were dispersed in simulated formation water at a concentration of 0.5 wt% and hydrated at 80 °C for 12 h to obtain hydrated gel particle suspensions. Before testing, the suspensions were gently stirred at 100 rpm for 5 min to ensure uniform particle dispersion and minimize the influence of sedimentation. Rheological measurements were conducted using an MCR 302 rotational rheometer (Anton Paar GmbH, Graz, Austria) equipped with a 25 mm parallel-plate geometry. The plate gap was fixed at 2.0 mm for all samples to avoid pre-compression of hydrated particles while maintaining stable particle distribution within the testing region. After the gap was set, excess sample around the plate edge was carefully removed, and a solvent trap was used to minimize water evaporation during high-temperature measurements.

During the measurement, strain sweep tests were first performed at a fixed frequency of 1 Hz to determine the linear viscoelastic region. Frequency sweep tests were then conducted within the linear viscoelastic region over a frequency range of 0.1–10 Hz. The storage modulus G′, loss modulus G″, and complex viscosity were recorded. Direct open-geometry rheological measurements were performed at 25 and 80 °C. To avoid water evaporation and boiling during high-temperature testing, the 120 °C rheological stability evaluation was conducted by sealed thermal aging rather than direct in situ measurement at 120 °C. Hydrated gel particle suspensions were sealed in high-temperature-resistant vessels and aged at 120 °C for the designed time. After aging, the samples were cooled to 80 °C and immediately measured under the same parallel-plate conditions. Thus, the G′ retention after 120 °C treatment represents the residual network strength after sealed thermal aging, rather than rheological data obtained under open boiling conditions. Each sample was tested in triplicate, and the results are expressed as mean ± standard deviation.

#### 4.4.4. Compression and Elastic Recovery Test

Uniaxial compression tests were performed to evaluate the compressive stability and elastic recovery of hydrated gel particles. Dry gel particles were hydrated in simulated formation water at 80 °C for 12 h, separated using a 100-mesh sieve, and gently blotted to remove surface free water. The hydrated particles were then packed into a cylindrical mold with a diameter of 20 mm and a height of 20 mm. The particle bed was slightly compacted to form a stable packed structure while avoiding excessive pre-compression that could damage the initial particle structure.

Compression tests were conducted using a universal testing machine at 25 °C. The compression rate was set at 1 mm/min, and the maximum compressive strain was set at 50%. The compressive stress–strain curves were recorded, and the compressive stress at 30% strain was used as an indicator of the compressive resistance of the particle packing. To evaluate elastic recovery, the samples were unloaded after being compressed to 50% strain and then allowed to stand for 10 min before measuring the recovered height. The height recovery ratio was calculated using Equation (2):

(2) $$R_{h}=\frac{H_{r}}{H_{0}}\times 100\%$$

where $R_{h}$ is the height recovery ratio, %; $H_{0}$ is the initial height of the particle packing before compression, mm; and $H_{r}$ is the recovered height after unloading and standing for 10 min, mm. Each test was repeated three times, and the results are expressed as mean ± standard deviation.

#### 4.4.5. Ester-Bond Cleavage and Self-Degradation Test

The ester-bond cleavage and self-degradation behavior of gel particles were evaluated by combining mass loss, particle size decay, rheological modulus variation, and Fourier transform infrared spectroscopy (FTIR). Specifically, 0.20 g of dry gel particles was added to 50 mL of simulated formation water and placed in sealed pressure-resistant reaction bottles for high-temperature degradation tests. The test temperatures were set at 80, 100, and 120 °C, and the degradation times were 6, 12, 24, 48, 72, and 96 h. To assess the influence of the aqueous chemical environment on ester hydrolysis, the pH values of the systems were adjusted to 5, 7, and 9.

After reaching the preset degradation time, the reaction bottles were removed and cooled to room temperature. The residual particles were collected using a 100-mesh sieve, rapidly rinsed with deionized water to remove surface salts, and dried at 45 °C to constant weight. The mass retention ratio of gel particles was calculated using Equation (3):

(3) $$M_{r}=\frac{m_{t}}{m_{0}}\times 100\%$$

where $M_{r}$ is the mass retention ratio, %; $m_{0}$ is the initial mass of dry gel particles, g; and $m_{t}$ is the dry mass of residual particles after degradation for time *t*, g.

The mass loss ratio was calculated using Equation (4):

(4) $$M_{l}=\frac{m_{0}-m_{t}}{m_{0}}\times 100\%$$

where $M_{l}$ is the mass loss ratio, %.

Particle-size changes before and after degradation were determined using the microscopic image analysis procedure described in Section 4.4.1. The G′ variation in degraded particle suspensions was measured according to the rheological protocol described in Section 4.4.3. To verify ester-bond cleavage, FTIR spectra of dried samples before and after degradation were recorded over a wavenumber range of 4000–500 cm^−1^ with a resolution of 4 cm^−1^ and 32 scans. The intensity changes in the ester carbonyl C=O stretching vibration peak and the C–O–C characteristic peak were compared to evaluate the influence of ester hydrolysis on the gel network structure. EGP-C was used as a nondegradable control to distinguish ester-bond-cleavage-induced network attenuation from structural changes caused by ordinary thermal aging. Each experiment was repeated three times.

#### 4.4.6. Single-Fracture Temporary Plugging Test

Single-fracture models were used to evaluate the temporary plugging ability of gel particles in fractures with different apertures. The experimental system consisted of a high-temperature and high-pressure holder, fractured cores or visual fracture plates, a constant-flow pump, a pressure sensor, and a liquid collection unit. The fracture apertures were set at 0.2, 0.5, 1.0, and 1.5 mm to simulate preferential flow channels with different scales. Before the test, gel particles with a particle size fraction of 300–600 μm were dispersed in simulated formation water at 0.5 wt% and hydrated at 80 °C for 12 h to obtain hydrated gel particle suspensions.

Before particle injection, the fracture model was pre-flushed with simulated formation water at a flow rate of 1.0 mL/min to remove trapped air and establish a stable initial pressure. The hydrated gel particle suspension was then injected at the same flow rate, and the inlet pressure and injected volume were continuously recorded. When the pressure reached a stable plateau or the injected particle suspension volume reached 1.0 PV, particle injection was stopped, and simulated formation water was continuously injected to evaluate the erosion resistance of the plugging structure. The breakthrough pressure, stable plugging pressure, outlet fluid state, and pressure fluctuation characteristics were recorded during the experiment.

The plugging efficiency was calculated using Equation (5):

(5) $$E_{p}=\frac{K_{0}-K_{p}}{K_{0}}\times 100\%$$

where $E_{p}$ is the plugging efficiency, %; $K_{0}$ is the equivalent permeability of the fracture model before plugging, mD; and $K_{p}$ is the equivalent permeability after plugging, mD.

The plugging pressure gradient was calculated using Equation (6):

(6) $$G_{p}=\frac{P_{b}}{L}$$

where $G_{p}$ is the plugging pressure gradient, MPa/m; P $P_{b}$ is the breakthrough pressure, MPa; and L is the length of the fracture model, m.

To analyze the size matching relationship between particles and fractures, the particle–fracture matching coefficient λ was defined as:

(7) $$\lambda =\frac{D_{50}}{W_{f}}$$

where λ is the particle–fracture matching coefficient; $D_{50}$ is the median diameter of hydrated gel particles, μm; and $W_{f}$ is the fracture aperture, μm. Each experiment was repeated three times.

#### 4.4.7. Multi-Fracture Selective Plugging and Flow Diversion Test

A parallel multi-fracture model was used to evaluate the selective plugging and flow diversion ability of gel particles in a multi-fracture system. The model consisted of three parallel fracture channels with apertures of 1.0, 0.5, and 0.2 mm, representing a dominant fracture, a medium fracture, and a narrow fracture, respectively. The inlets of the three fractures were connected to the same injection end, whereas the outlets were connected to independent measuring units to record the produced liquid volume and flow rate from each fracture channel in real time.

The experiment consisted of three stages. The first stage was initial water flooding, during which simulated formation water was injected at 1.0 mL/min, and the outlet flow rate of each fracture was recorded to calculate the initial flow split ratio. The second stage was gel particle temporary plugging, during which a 0.5 wt% hydrated gel particle suspension was injected into the parallel fracture model, and the inlet pressure and outlet flow rates of each fracture were continuously recorded. The third stage was subsequent water flooding, during which simulated formation water was continuously injected to evaluate the ability of fluid to divert from the plugged dominant fracture into medium and narrow fractures. The entire experiment was conducted at 80 °C, and each test was repeated three times.

The flow split ratio of each fracture channel was calculated using Equation (8):

(8) $$F_{i}=\frac{Q_{i}}{\sum Q_{i}}\times 100\%$$

where $F_{i}$ is the flow split ratio of the *i*-th fracture channel, %; $Q_{i}$ is the outlet flow rate of the *i*-th fracture channel, mL/min; and $\sum Q_{i}$ is the total outlet flow rate of all fractures, mL/min.

The flow diversion efficiency was calculated using Equation (9):

(9) $$E_{d}=\frac{F_{s,p}-F_{s,0}}{F_{d,0}}\times 100\%$$

where $E_{d}$ is the flow diversion efficiency, %; $F_{s,p}$ is the total flow split ratio of the medium and narrow fractures after plugging, %; $F_{s,0}$ is the total flow split ratio of the medium and narrow fractures during the initial water flooding stage, %; and $F_{d,0}$ is the initial flow split ratio of the dominant fracture, %.

#### 4.4.8. Controlled Deplugging, Flowback and Permeability Recovery Test

After the single-fracture or multi-fracture plugging tests, the controlled deplugging, flowback, and permeability recovery capacities of gel particles were further evaluated. The plugged fracture models were sealed and aged in a thermostatic environment using simulated formation water as the aging medium. The aging temperatures were set at 80, 100, and 120 °C, and the aging times were 12, 24, 48, 72, and 96 h. After reaching the preset aging time, the models were cooled to the experimental temperature and flushed with simulated formation water in either the forward or reverse direction at a flow rate of 1.0 mL/min. The inlet pressure, outlet liquid volume, and flowback residues were continuously recorded during the experiment.

The plugging pressure before deplugging was recorded as $P_{p}$, and the residual pressure after flushing to a stable state was recorded as $P_{r}$. The pressure recovery coefficient was calculated using Equation (10):

(10) $$R_{p}=\frac{P_{p}-P_{r}}{P_{p}}\times 100\%$$

where $R_{p}$ is the pressure recovery coefficient, %; $P_{p}$ is the plugging pressure before deplugging, MPa; and $P_{r}$ is the stable pressure after deplugging, MPa.

The permeability recovery ratio was calculated using Equation (11):

(11) $$R_{k}=\frac{K_{r}}{K_{0}}\times 100\%$$

where $R_{k}$ is the permeability recovery ratio, %; $K_{r}$ is the equivalent permeability of the fracture model after deplugging, mD; and $K_{0}$ is the initial equivalent permeability, mD.

The flowback ratio was calculated using Equation (12):

(12) $$R_{f}=\frac{m_{f}}{m_{i}}\times 100\%$$

where $R_{f}$ is the flowback ratio, %; $m_{i}$ is the mass of gel particles injected into the fracture model, g; and $m_{f}$ is the dry mass of gel particles and degradation residues collected from the flowback liquid, g.

To evaluate residual formation damage, the model was continuously flushed with simulated formation water until the pressure stabilized, and the permeability recovery of EGP-1, EGP-2, EGP-3, and EGP-C was compared. Each experiment was repeated three times.

#### 4.4.9. Morphological Characterization of Plugging and Degradation Residues

Optical microscopy, scanning electron microscopy (SEM), and microfocused X-ray computed tomography (μCT) were used to characterize the plugging morphology of gel particles and the features of degradation residues. After the visual fracture experiments, optical microscopy was used to directly observe the particle distribution near the fracture inlet, middle section, and outlet. These observations were used to identify particle migration, bridging, accumulation, and local erosion characteristics. For fractured core models, the samples were cut along the fracture direction after the experiment, and residual particles from the inlet, middle, and outlet regions were collected for observation.

SEM was used to observe the surface morphology of dry gel particles, freeze-dried hydrated particles, and degradation residues. Hydrated samples were freeze-dried at −50 °C for 24 h, fixed onto conductive adhesive tape, and sputter-coated with gold. The surface pores, network collapse, and degradation-induced fragmentation features of the particles were observed using SEM at an accelerating voltage of 5 kV.

When necessary, μCT was used to perform three-dimensional scanning and reconstruction of the plugged fracture models. Before scanning, the fracture models were sealed and fixed, and the scanning resolution was set to 5–10 μm/voxel according to the fracture scale. Grayscale threshold segmentation was used to distinguish the fracture space, rock matrix, and particle retention region. The plugging zone length, particle accumulation volume fraction, and changes in fracture connectivity were extracted to support the analysis of the spatial plugging morphology of gel particles within fractures.

#### 4.4.10. Data Processing and Statistical Analysis

All experiments were performed at least in triplicate, and the results are expressed as mean ± standard deviation. Particle size distribution, swelling ratio, mass loss ratio, plugging efficiency, flow diversion efficiency, pressure recovery coefficient, flowback ratio, and permeability recovery ratio were calculated based on repeated experimental results. Differences among sample groups were analyzed using one-way analysis of variance followed by Tukey’s post hoc test for multiple comparisons. For comparisons involving different formulations, fracture widths, temperatures, or salinities, statistical significance was evaluated at the same testing condition or at the selected endpoint shown in the corresponding figure. A value of *p* < 0.05 was considered statistically significant. In the revised figures and tables, different lowercase letters indicate statistically significant differences among groups, whereas groups sharing the same letter are not significantly different.

Before plotting, the completeness of the raw data was checked. Outliers were excluded only when they could be clearly attributed to abnormal instrument acquisition, leakage, plugging failure, or operational error, and the original records were retained for traceability. All pressure, flow rate, and mass data were maintained with corresponding raw records and calculated results to ensure the traceability of the data processing procedure.

## Figures and Tables

**Table 1:** Main FTIR band assignments.

Wavenumber/cm^−1^	Main Assignment	Structural Indication
3420	N–H/O–H stretching	Amide groups, hydrogen-bonded water, and hydrophilic network
1655	Amide I C=O stretching	AAm-based backbone structure
1548	Amide II N–H/C–N vibration	Amide- and NVP-related structures
1202	S=O stretching	AMPS-derived sulfonic groups
1125	C–O–C stretching	PEGDA incorporation into the crosslinked network
1700–1740	Ester carbonyl C=O weak shoulder/enhanced absorption	PEGDA-derived ester-containing crosslinks

**Table 2:** Flow diversion efficiency of EGP-2 in the multi-fracture system.

Sample	Flow Diversion Efficiency Mean/%	SD
EGP-2	58.2	2.7

**Table 3:** Selective plugging and flow diversion results of different gel particles in the multi-fracture system.

Sample	Dominant Fracture Flow Split/%	Medium Fracture Flow Split/%	Narrow Fracture Flow Split/%	Flow Diversion Efficiency/%
EGP-1	45.3 ± 2.4	35.1 ± 1.8	19.6 ± 1.3	42.2 ± 2.9
EGP-2	32.8 ± 1.9	43.6 ± 2.0	23.6 ± 1.4	58.2 ± 2.7
EGP-3	38.6 ± 2.1	40.4 ± 1.9	21.0 ± 1.3	50.8 ± 2.5
EGP-C	36.4 ± 2.0	40.9 ± 1.8	22.7 ± 1.2	53.6 ± 2.6

Note: Data are expressed as mean ± standard deviation, *n* = 3.

**Table 4:** Comparison of EGP-2 with representative degradable gel particle systems and temporary plugging materials.

System	Main Evaluation Focus	Swelling Behavior	Plugging Performance	Degradation Behavior	Recovery/Residue Performance	Main Distinction from This Work
Degradable preformed particle gel reported by Zhu et al. [12]	Temporary plugging and permeability recovery in porous media	Static swelling and self-degradation were evaluated	Temporary plugging efficiency > 94%	Self-degradation under reservoir conditions	Permeability recovery > 90%	Mainly focused on porous-medium plugging and recovery; multi-fracture flow redistribution was not the central evaluation
Crosslinker-molecular-weight-regulated DPPG reported by Zhang et al. [14]	Effect of crosslinker molecular weight on swelling and degradation	Swelling ratio of approximately 7–33 times	Temporary plugging behavior in low-temperature reservoir conditions	Complete degradation time of approximately 40–210 min	Damage reduction after degradation was evaluated	Emphasized swelling–degradation regulation by PEGDA molecular weight; selective flow diversion and flowback residue control were less directly addressed
Present EGP-2 system	Plugging–diversion–deplugging in a heterogeneous multi-fracture model	Equilibrium swelling ratio of 7.12 g/g at 80 °C and 100,000 mg/L salinity	Plugging efficiencies of 98.6% and 97.4% in 0.5 and 1.0 mm fractures	Mass loss ratio of 78.4% after 96 h at 120 °C	Flowback ratio of 82.6%, permeability recovery ratio of 88.7%, and residue ratio of 10.9%	Integrates fracture-scale plugging, multi-fracture flow redistribution, controlled degradation, and low-residue recovery

**Table 5:** Materials used in this study.

Material	Specification	Supplier	Role in System
Acrylamide (AAm)	≥99.0%	Aladdin Biochemical Technology Co., Ltd., Shanghai, China	Main monomer
2-Acrylamido-2-methylpropanesulfonic acid (AMPS)	≥98.0%	Macklin Biochemical Co., Ltd., Shanghai, China	Salt-tolerant functional monomer
N-Vinyl-2-pyrrolidone (NVP)	≥99.0%	Macklin Biochemical Co., Ltd., Shanghai, China	Thermally stabilizing auxiliary monomer
Poly(ethylene glycol) diacrylate (PEGDA)	Mn = 575 g/mol	Aladdin Biochemical Technology Co., Ltd., Shanghai, China	Hydrolysable ester-bond-containing crosslinker
N,N′-Methylenebisacrylamide (MBAA)	≥99.0%	Aladdin Biochemical Technology Co., Ltd., Shanghai, China	Nondegradable control crosslinker
Ammonium persulfate (APS)	≥98.0%	Sinopharm Chemical Reagent Co., Ltd., Shanghai, China	Oxidizing initiator
Sodium bisulfite (NaHSO_3_)	≥98.0%	Xilong Scientific Co., Ltd., Shantou, China	Reducing initiator
Sodium hydroxide (NaOH)	≥96.0%	Xilong Scientific Co., Ltd., Shantou, China	pH adjustment
Sodium chloride (NaCl)	≥99.5%	Xilong Scientific Co., Ltd., Shantou, China	Simulated formation water component
Calcium chloride (CaCl_2_)	≥96.0%	Macklin Biochemical Co., Ltd., Shanghai, China	Simulated formation water component
Magnesium chloride hexahydrate (MgCl_2_·6H_2_O)	≥98.0%	Aladdin Biochemical Technology Co., Ltd., Shanghai, China	Simulated formation water component
Deionized water	Resistivity ≥ 18.2 MΩ·cm	Laboratory purification system	Solvent and washing medium
Nitrogen gas (N_2_)	≥99.99%	Chengdu Taiyu Industrial Gases Co., Ltd., Chengdu, China	Oxygen removal during polymerization
Standard fracture plates	0.2, 0.5, 1.0, and 1.5 mm apertures	Jiangsu Hai’an Petroleum Scientific Instrument Co., Ltd., Hai’an, China	Single-fracture plugging medium
Three-parallel-fracture model	1.0, 0.5, and 0.2 mm parallel fractures	Jiangsu Hai’an Petroleum Scientific Instrument Co., Ltd., Hai’an, China	Multi-fracture flow diversion medium

**Table 6:** Major instruments and testing systems.

Instrument	Model	Manufacturer	Application
Fourier transform infrared spectrometer	Nicolet iS50	Thermo Fisher Scientific, Waltham, MA, USA	Chemical structure analysis
Scanning electron microscope	JSM-IT500	JEOL Ltd., Tokyo, Japan	Particle surface morphology observation
Optical microscope	DM2700 M	Leica Microsystems, Wetzlar, Germany	Particle size observation and image acquisition
Laser particle size analyzer	Mastersizer 3000	Malvern Panalytical, Malvern, UK	Particle size distribution measurement
Rotational rheometer	MCR 302 with 25 mm parallel-plate geometry	Anton Paar GmbH, Graz, Austria	Rheological characterization of hydrated gel particle suspensions
Universal testing machine	CMT5105	MTS Industrial Systems Co., Ltd., Shenzhen, China	Compression and elastic recovery tests
High-temperature drying oven	DHG-9070A	Shanghai Yiheng Scientific Instrument Co., Ltd., Shanghai, China	Gel drying and sample pretreatment
Thermostatic water bath	HH-6	Changzhou Guohua Electric Appliance Co., Ltd., Changzhou, China	Polymerization and hydration temperature control
High-temperature aging reactor	WHF-100	Jiangsu Hai’an Petroleum Scientific Instrument Co., Ltd., Hai’an, China	Degradation and aging tests
High-temperature fracture plugging system	HTHP-FP100	Jiangsu Hai’an Petroleum Scientific Instrument Co., Ltd., Hai’an, China	Single-fracture plugging and deplugging tests
Parallel-fracture flow diversion system	PFM-3	Jiangsu Hai’an Petroleum Scientific Instrument Co., Ltd., Hai’an, China	Multi-fracture selective plugging and flow diversion tests
High-precision syringe pump	LSP01-1A	Baoding Longer Precision Pump Co., Ltd., Baoding, China	Controlled injection of particle suspension
Pressure transducer	CYB-20S	Beijing Star Sensor Technology Co., Ltd., Beijing, China	Real-time pressure monitoring
Electronic balance	BSA224S	Sartorius, Göttingen, Germany	Mass measurement
pH meter	PHS-3C	Shanghai INESA Scientific Instrument Co., Ltd., Shanghai, China	pH adjustment and monitoring
Standard sieve shaker	ZBSX-92A	Zhejiang Shangyu Huafeng Instrument Co., Ltd., Shaoxing, China	Gel particle sieving

**Table 7:** Formulation of ester-bond-cleavable self-degradable gel particles.

Sample	AAm/g	AMPS/g	NVP/g	PEGDA/g	MBAA/g	APS/g	NaHSO_3_/g	Crosslinking Feature
EGP-1	9.00	3.75	2.25	0.15	—	0.10	0.10	Low ester-bond crosslinking density
EGP-2	9.00	3.75	2.25	0.30	—	0.10	0.10	Moderate ester-bond crosslinking density
EGP-3	9.00	3.75	2.25	0.45	—	0.10	0.10	High ester-bond crosslinking density
EGP-C	9.00	3.75	2.25	—	0.045	0.10	0.10	Nondegradable covalent crosslinking

**Table 8:** Dry-gel yield and target particle-fraction yield of different gel particle formulations.

Sample	Crosslinker Condition	Theoretical Solid Feed/g	Dry Gel Mass/g	Dry-Gel Yield/%	300–600 μm Particle Mass/g	Target Particle-Fraction Yield/%
EGP-1	PEGDA, 1.0 wt%	15.35	13.61 ± 0.18	88.7 ± 1.2	5.86 ± 0.15	43.1 ± 1.4
EGP-2	PEGDA, 2.0 wt%	15.50	13.93 ± 0.16	89.9 ± 1.0	7.18 ± 0.17	51.5 ± 1.3
EGP-3	PEGDA, 3.0 wt%	15.65	14.21 ± 0.14	90.8 ± 0.9	8.12 ± 0.19	57.1 ± 1.2
EGP-C	MBAA, 0.30 wt% relative to monomers	15.245	13.85 ± 0.13	90.8 ± 0.8	8.24 ± 0.18	59.5 ± 1.1

Note: Theoretical solid feed includes AAm, AMPS, NVP, crosslinker, APS, and NaHSO_3_. Data are expressed as mean ± standard deviation, *n* = 3.

## Data Availability

The data presented in this study are available on request from the corresponding author.

## References

[1] Seright R.S., Lane R.H., Sydansk R.D. (2003). A Strategy for Attacking Excess Water Production. SPE Prod. Facil..

[2] Kang W., Kang X., Lashari Z.A., Li Z., Zhou B., Yang H., Sarsenbekuly B., Aidarova S. (2021). Progress of Polymer Gels for Conformance Control in Oilfield. Adv. Colloid Interface Sci..

[3] Zhu D., Bai B., Hou J. (2017). Polymer Gel Systems for Water Management in High-Temperature Petroleum Reservoirs: A Chemical Review. Energy Fuels.

[4] de Aguiar K.L.N.P., de Oliveira P.F., Mansur C.R.E. (2020). A Comprehensive Review of In Situ Polymer Hydrogels for Conformance Control of Oil Reservoirs. Oil Gas Sci. Technol. Rev. IFP Energ. Nouv..

[5] Seidy Esfahlan M., Khodapanah E., Tabatabaei-Nezhad S.A. (2021). Comprehensive Review on the Research and Field Application of Preformed Particle Gel Conformance Control Technology. J. Pet. Sci. Eng..

[6] Bai B., Li L., Liu Y., Liu H., Wang Z., You C. (2007). Preformed Particle Gel for Conformance Control: Factors Affecting Its Properties and Applications. SPE Reserv. Eval. Eng..

[7] Bai B., Liu Y., Coste J.-P., Li L. (2007). Preformed Particle Gel for Conformance Control: Transport Mechanism through Porous Media. SPE Reserv. Eval. Eng..

[8] Seright R.S. (2001). Gel Propagation through Fractures. SPE Prod. Facil..

[9] Song Z., Bai B., Zhang H. (2018). Preformed Particle Gel Propagation and Dehydration through Semi-Transparent Fractures and Their Effect on Water Flow. J. Pet. Sci. Eng..

[10] Wang Z., Bai B. (2018). Preformed-Particle-Gel Placement and Plugging Performance in Fractures with Tips. SPE J..

[11] Wang Z., Bai B., Sun X., Wang J. (2019). Effect of Multiple Factors on Preformed Particle Gel Placement, Dehydration, and Plugging Performance in Partially Open Fractures. Fuel.

[12] Zhu D.Y., Fang X.Y., Sun R.X., Xu Z.H., Liu Y., Liu J.Y. (2021). Development of Degradable Pre-Formed Particle Gel (DPPG) as Temporary Plugging Agent for Petroleum Drilling and Production. Pet. Sci..

[13] Zhu D., Xu Z., Sun R., Fang X., Gao D., Jia X., Hu J., Weng J. (2021). Laboratory Evaluation on Temporary Plugging Performance of Degradable Preformed Particle Gels (DPPGs). Fuel.

[14] Zhang H.J., Zhu D.Y., Gong Y.L., Qin J.H., Liu X.N., Pi Y.H., Zhao Q., Luo R.T., Wang W.S., Zhi K.K. (2022). Degradable Preformed Particle Gel as Temporary Plugging Agent for Low-Temperature Unconventional Petroleum Reservoirs: Effect of Molecular Weight of the Cross-Linking Agent. Pet. Sci..

[15] Zhao S., Zhu D., Bai B. (2021). Experimental Study of Degradable Preformed Particle Gel (DPPG) as Temporary Plugging Agent for Carbonate Reservoir Matrix Acidizing to Improve Oil Recovery. J. Pet. Sci. Eng..

[16] Zhang L., Zhou F., Mou J., Pournik M., Tao S., Wang D., Wang Y. (2019). Large-Scale True Tri-Axial Fracturing Experimental Investigation on Diversion Behavior of Fiber Using 3D Printing Model of Rock Formation. J. Pet. Sci. Eng..

[17] Zhang L., Zhou F., Mou J., Feng W., Li Z., Zhang S. (2020). An Integrated Experimental Method to Investigate Tool-Less Temporary-Plugging Multistage Acid Fracturing of Horizontal Well by Using Self-Degradable Diverters. SPE J..

[18] Zhao L., Chen X., Zou H., Liu P., Liang C., Zhang N., Li N., Luo Z., Du J. (2020). A Review of Diverting Agents for Reservoir Stimulation. J. Pet. Sci. Eng..

[19] Tessarolli F.G.C., Gomes A.S., Mansur C.R.E. (2019). Gelation Kinetics of Hydrogels Based on Acrylamide–AMPS–NVP Terpolymer, Bentonite, and Polyethylenimine for Conformance Control of Oil Reservoirs. Gels.

[20] Stillman Z.S., Jarai B.M., Raman N., Patel P., Fromen C.A. (2020). Degradation Profiles of Poly(ethylene glycol) Diacrylate (PEGDA)-Based Hydrogel Nanoparticles. Polym. Chem..

[21] Zustiak S.P., Leach J.B. (2010). Hydrolytically Degradable Poly(ethylene glycol) Hydrogel Scaffolds with Tunable Degradation and Mechanical Properties. Biomacromolecules.

[22] Rodriguez-Rivera G.J., Green N.H., Kloxin A.M., Burdick J.A., Zustiak S.P. (2024). A User’s Guide to Degradation Testing of Polyethylene Glycol-Based Hydrogels: From In Vitro to In Vivo Studies. J. Biomed. Mater. Res. A.

